# Pneumothorax in COVID-19 Acute Respiratory Distress Syndrome: Case Series

**DOI:** 10.7759/cureus.11749

**Published:** 2020-11-28

**Authors:** Kelvin Wong, Dae Hyeon Kim, Annamaria Iakovou, Sameer Khanijo, Adey Tsegaye, Stella Hahn, Mangala Narasimhan, Gulrukh Zaidi

**Affiliations:** 1 Pulmonary and Critical Care Medicine, Northwell Health North Shore University Hospital, Manhasset, USA; 2 Pulmonary and Critical Care Medicine, Long Island Jewish Medical Center, Manhasset, USA; 3 Pulmonary and Critical Care Medicine, Donald and Barbara Zucker School of Medicine at Hofstra/Northwell, New York, USA

**Keywords:** covid-19, severe acute respiratory syndrome coronavirus 2, respiratory distress syndrome, pneumothorax, lung compliance

## Abstract

Objective

The study aims to describe the clinical characteristics and outcomes of patients with COVID-19 related acute respiratory distress syndrome (ARDS) who developed pneumothorax.

Design and setting

A retrospective chart review was performed of the electronic medical record. Patients were included if they were identified as having confirmed COVID-19 as well as pneumothorax from March 16, 2020 to May 31, 2020. Patients' demographic and clinical characteristics, mechanical ventilator parameters, lung compliance measurements and outcomes during hospitalization were collected. This case series was conducted in intensive care units at two large tertiary care centers within the Northwell Health System, located in New York State.

Patients

A total of 75 patients were identified who were predominantly male (73.3%) with an average age of 62.8 years. Thirty (40%) were Hispanic, 20 (26.7%) were White, 16 (21.3%) were Asian, and nine (12%) were Black. Common comorbid conditions were hypertension (52%), diabetes mellitus (26.7%), hyperlipidemia (32.0%), and chronic pulmonary disease (8, 10.7%).

Measurements and main results

Most of the patients were diagnosed with pneumothorax while on mechanical ventilation (92%) despite overall adherence with lung-protective ventilation strategies. Average tidal volume was 6.66 mL/kg) of ideal body weight. The average positive end-expiratory pressure (PEEP) was 10.83 (cm) H2O. Lung compliance was poor, with average peak and plateau pressures of 41.9 cm H2O and 35.2 cm H2O, respectively. Inpatient mortality was high in these patients (76%). Conservative management with initial observation had a success rate (73.3%) with similar mortality and shorter length of stay (LOS) on average. Significant factors in the conservatively managed group included lack of tension physiology, the smaller size of pneumothorax, lack of underlying diabetes, presence of pneumomediastinum, and not being on mechanical ventilation during diagnosis.

Conclusion

Despite overall adherence to best practice ventilator management in ARDS, we observed a large number of pneumothoraces during the COVID-19 pandemic. Conservative management may be appropriate if there are no clinical signs or symptoms of tension physiology and pneumothorax size is small.

## Introduction

Coronavirus disease 2019 (COVID-19) secondary to severe acute respiratory syndrome coronavirus-2 (SARS-CoV-2) inundated the New York metropolitan area from March to May 2020. During this time period, our health system treated over 17,000 patients with COVID-19, of which approximately 3,000 required mechanical ventilation. While these patients presented with a wide range of illnesses, most of the intubated patients had acute respiratory distress syndrome (ARDS).

Patients with ARDS are known to be at risk for pulmonary barotrauma during mechanical ventilation, which is associated with increased morbidity and mortality [1]. The mechanism involves elevated transpulmonary pressures, alveolar rupture, and release of air into the extra-alveolar space, which can cause pneumothorax, pneumomediastinum, and subcutaneous emphysema. The incidence of pulmonary barotrauma in mechanically ventilated patients depends on the underlying disease process and can range from 0-50% [2]. In ARDS, the overall incidence of barotrauma is 6.5%, with a relative risk of 2.7 likely related to the low compliance of the respiratory system in this disease state [3]. The risk of barotrauma can be reduced by implementing lung-protective ventilation strategies [4]. Prior studies have shown that the use of high tidal volumes and plateau pressures resulted in a 42% rate of pneumothorax and 71% mortality rate compared with 7% and 38%, respectively, in patients in whom tidal volumes and plateau pressures were limited [5].

In the 2003 severe acute respiratory syndrome coronavirus-1 (SARS-CoV-1) epidemic, spontaneous pneumomediastinum and pneumothorax were also reported [6]. Observational studies reported the occurrence of pneumothorax in 20-34% of patients with SARS-CoV-1 who required mechanical ventilation [7, 8]. There have been case reports of pneumothorax associated with COVID-19 ARDS, as well as a recently published multicenter retrospective case series in the United Kingdom [9, 10]. Here we report the largest case series of pneumothorax in this patient population and describe their clinical characteristics and outcomes.

## Materials and methods

Cases were collected retrospectively based on author recall. Data was collected from the electronic medical record (EMR), Enterprise SunriseClinical Manager™ - Allscripts®, for patients with confirmed COVID-19 and pneumothorax admitted to North Shore University Hospital and Long Island Jewish Medical Center, two large tertiary care centers within the Northwell Health System, from March 16, 2020 to May 31, 2020. The Northwell Health System is New York State’s largest healthcare provider, consisting of a total of 23 hospitals. This case series was approved by the Northwell Institutional Review Board (IRB) as minimal-risk research using data collected for routine clinical practice and waived the requirement for informed consent (IRB number 20-0200).

A confirmed case of COVID-19 was defined as a positive laboratory test for SARS-CoV-2 from real-time reverse transcription-polymerase chain reaction (RT-PCR) assay of the respiratory specimen. Pneumothorax was identified by chest radiograph or point of care ultrasound (POCUS). The presence of tension pneumothorax was based on documentation in the EMR as determined by the clinical team. Size of pneumothorax was measured on upright chest radiograph when available, as the distance (mm) from lung apex to the top margin of the visceral pleura. We collected data on patient demographics, baseline comorbidities, invasive procedures, ventilator parameters shortly prior to pneumothorax diagnosis, length of hospital stay prior to pneumothorax, days on mechanical ventilation prior to pneumothorax and, patient outcomes (extubation, tracheostomy, decannulation, hospital length of stay, and in-hospital mortality).

## Results

A total of 75 patients with COVID-19 and pneumothorax were identified. The average age was 62.8 years (range: 25-90). Thirty (40%) were Hispanic, 20 (26.7%) were White, 16 (21.3%) were Asian, and nine (12%) were Black. Patients were predominantly male (55, 73.3%). Forty-eight patients (68.6%) had a normal body mass index (BMI 18-25), 22 (29.3%) were obese (BMI>30), and two (2.7%) were morbidly obese (BMI>40). Comorbid conditions included hypertension (39, 52%), diabetes mellitus (20, 26.7%), hyperlipidemia (24, 32.0%), and chronic pulmonary disease (8, 10.7%). Fifteen patients (20.0%) had no past medical history (Table 1).

**Table 1:** Patients' demographic and clinical characteristics Values are expressed as number (percentage) unless otherwise indicated. * Close observation strategy ** Immediate chest tube placement HTN - hypertension; HLD - hyperlipidemia; DM - diabetes mellitus; COPD - chronic obstructive pulmonary disease; PTX - pneumothorax

Demographics	Total cohort (n=75)	Conservative* (n=15)	Invasive** (n=60)	p-value
Age (years)				
Mean [Range]	62.8 [25 – 90]	63.67	62.13	0.682
Gender				
Male	55 (73.3)	9 (60)	46 (76.7)	0.205
Female	20 (26.7)	6 (40)	14 (23.3)	0.205
BMI (kg/m2)				
Mean [Range]	28.6 [16.1 - 42.2]	27.7	28.8	0.472
Underweight	1 (1.3)	0 (0)	1 (1.7)	0.693
Normal	48 (68.6)	11 (73.3)	37 (61.7)	0.975
Obese	22 (29.3)	2 (13.3)	20 (33.3)	0.149
Morbidly obese	2 (2.7)	1 (6.7)	1 (1.7)	0.322
Not reported	2 (2.7)	1 (6.7)	1 (1.7)	
Race				
Hispanic	30 (40)	6 (40)	24 (40)	1.000
White	20 (26.7)	5 (33.3)	15 (25)	0.528
Asian	16 (21.3)	3 (20)	13 (21.7)	0.890
Black	9 (12)	1 (6.7)	8 (13.3)	0.486
Comorbidities				
HTN	39 (52)	6 (40)	33 (55)	0.302
HLD	24 (32)	4 (26.7)	20 (33.3)	0.626
DM	20 (26.7)	1 (6.7)	19 (31.7)	0.049
COPD	8 (10.6)	0 (0)	8 (13.3)	0.138
None	15 (20)	5 (33.3)	10 (16.7)	0.153
P/F Ratio				
Mild (200-300)	12 (16)	2 (13.3)	10 (16.7)	0.749
Moderate (100-200)	31 (41.3)	7 (46.7)	24 (40)	0.639
Severe (<100)	32 (42.7)	6 (40)	26 (43.3)	0.818
Other characteristics				
On paralytics	23 (30.7)	3 (20)	20 (33.3)	0.321
Procedure within 24 hours prior	32 (42.6)	6 (40)	26 (43.3)	0.818
Presence of tension	29 (38.7)	0 (0)	29 (48.3)	<0.001
On mechanical ventilation	69 (92)	11 (73.3)	58 (96.7)	0.003
Pneumomediastinum also present	27 (36)	10 (66.7)	17 (28.3)	0.006
Average PTX size (mm)	35.82	10.23	44.17	<0.001

All pneumothoraces were diagnosed by chest radiograph, POCUS, or a combination of both. Upright chest radiograph at time of diagnosis was available in 61 patients (81.3%). Fifty-two patients had a unilateral pneumothorax, 31 right-sided, and 21 left-sided. Twenty-three patients had bilateral pneumothoraces. Of these, four patients initially had unilateral involvement and subsequently developed a contralateral pneumothorax. The average size of pneumothorax was 10.2 mm in the conservative group (close observation strategy, no immediate chest tube placement) and 44.2 mm in the invasive group (immediate chest tube placement). The average size of pneumothorax overall was 35.8 mm. Additionally, 27 patients (36.0%) also had pneumomediastinum (Table 1).

Sixty patients (80%) had small-bore (< 20 French), pigtail chest tubes placed immediately after pneumothorax diagnosis. Of these, 29 patients (48.3%) had clinical signs of tension (Table 1). Fifteen patients (20%) were managed conservatively (close observation strategy, no immediate chest tube placement), of which four patients (26.7%) ultimately failed, requiring chest tube placement 2.5 days on average after diagnosis (range: 1 to 6 days). Of the four patients who failed conservative management, the average size of pneumothorax was 18.7 mm (3 mm, 9.8mm, 15 mm, 47 mm). Indications for chest tube placement for those who failed observation were pneumothorax enlargement on serial chest radiographs (two patients), worsening tachypnea (one patient), and development of severe subcutaneous emphysema with elevated ventilator peak pressures after intubation (one patient). None of those who failed conservative management had hemodynamic compromise prior to chest tube placement. Overall, 64 patients (85.3%) required chest tube placement, while 11 (14.7%) did not.

Thirty-three patients (44%) had a procedure performed within 24 hours of pneumothorax identification. The most common procedures performed were intubation (15 patients, 45.5%) and central line placement (six patients, 18.2%). Both procedures were performed in eight patients (24.2%). Additionally, pneumothoraces were found in two patients following cardiopulmonary resuscitation (CPR) and two after tracheostomy (Table 2).

**Table 2:** Patients' clinical data *Close observation strategy **Immediate chest tube placement IBW - ideal body weight; PEEP - positive end-expiratory pressure

Clinical data	Total cohort (n=75)	Conservative* (n=15)	Invasive** (n=60)	p-value
Day of hospitalization at time of pneumothorax				
Mean [Range]	14.72 [0 – 63]	11	15.7	0.186
Days on ventilator prior to pneumothorax				
Mean [Range]	7.57 [0 – 47]	4.4	8.2	0.191
Tidal volume (mL/kg of IBW)				
Mean (SD)	6.66 (1.08)	6.80 (1.29)	6.63 (1.05)	0.569
Peak pressure (cm H2O)				
Mean (SD)	41.86 (8.13)	44.18 (9.29)	41.4 (7.88)	0.242
Plateau pressure (cm H20)				
Mean (SD)	35.17 (8.19)	38.0 (7.55)	34.48 (9.94)	0.204
PEEP (cm H2O)				
Mean (SD)	10.83 (4.19)	10.36 (3.11)	10.93 (4.39)	0.644
Outcome (n,%)				
Inpatient deaths	57 (76)	11 (73.3)	46 (76.7)	0.788
Extubated	4 (5.3)	2 (13.3)	2 (3.3)	0.126
Tracheostomy	15 (21.7)	1 (6.7)	14 (23.3)	0.152
Decannulated	3 (4)	0 (0)	3 (16.7)	0.667
Discharged	18 (24)	4 (26.7)	14 (23.3)	0.786

Sixty-nine patients (92%) were receiving mechanical ventilation at the time of pneumothorax identification. The average time in the hospital was 14.7 days, and the average time on mechanical ventilation was approximately 7.5 days prior to pneumothorax identification. The average tidal volume was 6.66 milliliters per kilogram (mL/kg) of ideal body weight (IBW) with a range of 3.16 to 8.59 mL/kg of IBW. The average positive end-expiratory pressure (PEEP) was 10.83 centimeters (cm) H2O ± 4.19. The average peak and plateau pressures were 41.9 cm H2O and 35.2 cm H2O, respectively (Table 2).

Twenty-seven patients received CT of the chest in the week prior to the identification of pneumothorax. The majority showed diffuse bilateral ground-glass and consolidative opacities, while a few showed cystic changes and traction bronchiectasis.

At the time of analysis, 57 patients (76%) died, and 18 patients (24%) were discharged. No patients remained endotracheally intubated; 15 patients (21.7%) underwent a tracheostomy, of which three were later decannulated. The average time from identification of pneumothorax to death was 9.0 days. The overall average length of stay (LOS) for discharged patients was 48.7 days, with the conservative group average LOS being 37.5 days, and the invasive group average LOS being 51.3 days (Table 2).

## Discussion

In our COVID-19 experience, we noticed a seemingly higher incidence of pneumothorax in ARDS than expected compared to our pre-COVID-19 experience. Here, we looked at a total of 75 cases identified based on author recall at two large tertiary hospitals. While we are unable to extrapolate the incidence of pneumothorax in COVID-19 ARDS based on this, we were able to obtain data from a larger section of the Northwell Health system, which demonstrated a pneumothorax incidence of 13.72% for 1,822 COVID-19 ARDS patients. For comparison, prior to COVID-19, the overall incidence of barotrauma in ARDS was noted to be 6.5% [3]. 

The majority of patients in our case series were males older than 60 years with pre-existing conditions such as diabetes, hypertension, and obesity, all of which are associated with increased severity of illness in COVID-19 [11]. In our series, 76% of patients who developed a pneumothorax died. In comparison, data from across the entire Northwell Health system, from the beginning of the COVID-19 pandemic to date, shows a 68% mortality rate for 2,468 COVID-19 ARDS patients. This increased mortality signal suggests that the occurrence of pneumothorax may be a marker of disease severity and mortality. 

Our findings are different from the recently published ERS case series, which demonstrated a 63.1% overall survival of COVID-19 patients with pneumothorax [10]. They concluded that their data did not support previous suggestions that the development of pneumothorax is a grave prognostic marker. This difference may be due to different patient populations as only 63.3% of their patients required invasive ventilatory support compared to 92% of the patients in our case series. The majority of our patients had moderate or severe ARDS (84%) (Table 1). We were unable to compare ARDS severity as it was not reported in their case series. 

ARDS of any etiology is known to cause pathophysiologic alterations in the lungs that increase pneumothorax risk [5]. Studies have shown that the implementation of lung-protective strategies during mechanical ventilation leads to a significant reduction in this risk [5]. Ninety-two percent of the patients in our case series were on mechanical ventilation at the time of pneumothorax identification. The average tidal volume and PEEP were within the acceptable range for lung-protective ventilation. Ventilator dyssynchrony is also known to contribute to barotrauma. In our case series, 69% of patients were not on paralytics at the time of pneumothorax identification. It is unknown if ventilator dyssynchrony played a significant role as the majority of patients were heavily sedated. 

The average length of hospitalization prior to pneumothorax identification was 14 days, and the average time on mechanical ventilation was approximately 7.5 days prior to pneumothorax identification. Despite the implementation of lung-protective ventilation strategies, high average peak and plateau pressures were observed. This suggests disease progression and increased severity with subsequently reduced lung compliance prior to the eventual occurrence of pneumothorax. 

Forty-four percent of patients had a procedure prior to pneumothorax identification, with intubation being the most common. In our pre-COVID-19 experience at our hospitals, which are major referral centers for acute lung injury, pneumothorax is a very rare complication of intubation. This raises the question of whether these pneumothoraces were present prior to intubation. Additionally, six patients developed a spontaneous pneumothorax without any positive pressure ventilation, which further supports that COVID-19 itself may predispose patients to the development of pneumothorax. 

Conservative management (no immediate chest tube placement) had a high success rate (73.3%) in our study despite similar disease severity as defined by Berlin criteria for ARDS. While there was no significant difference in mortality, the conservative group had significantly shorter average LOS for discharged patients (37.5 days vs. 51.3 days, p=0.05). This may be explained by comparing the average time to pneumothorax resolution in the conservative group (9.3 days) compared to the average time to chest tube removal in the invasive group (18 days). This is likely due to hesitancy in removing chest tubes once placed, as there have been case reports of both refractory and recurrent pneumothorax in COVID-19. Recurrent pneumothorax was not observed after chest tube removal in our case series. While there were no chest tube related complications, this data may support a conservative strategy of watchful waiting when appropriate. 

Clinical signs of tension physiology provide a clear indication for immediate intervention, present in 29 patients (38.7%) of the invasive group compared to 0 patients (0%) of the conservative group (p=0.001). Absent signs of tension, pneumothorax size may be able to guide clinicians in choosing a treatment strategy. The average size of pneumothorax was 10.2 mm in the conservative group and 44.2 mm in the invasive group (p<0.001). Given the high success rate of conservative management and similar outcomes other than shorter average LOS, conservative management in the instance of small pneumothorax may be appropriate. Three of the four patients who failed conservative management had relatively large pneumothoraces (9.8 mm, 15 mm, 47 mm), supporting our findings. 

Not being on mechanical ventilation at the time of pneumothorax diagnosis and lack of diabetes as comorbidity may also support opting for a conservative approach. Those who were conservatively managed were less likely to be on mechanical ventilation at the time of diagnosis (p=0.003) and less likely to have underlying diabetes (p=0.049). The concurrent presence of pneumomediastinum at pneumothorax does not seem to indicate a greater need for intervention as the conservative group was more likely to have pneumomediastinum at pneumothorax diagnosis (p<0.001). 

Limitations

At the peak of the COVID-19 pandemic in New York, the overwhelming number of patients led to the creation of surge intensive care units (ICUs). Some of these were staffed by non-critical care physicians, nurses, and respiratory therapists that were deployed from other areas of the health system as well as other states. This led to variability in ventilator management and documentation in the EMR. 

Several limitations are inherent as this was a retrospective chart review that was dependent on the available documentation. It was difficult to determine ventilator dyssynchrony, adequacy of sedation, and any ventilator parameter adjustments that were not documented. The lack of real-time data on lung compliance and clinical context hinders identifying factors that may have led to pneumothorax development. If available, this may have allowed for differentiation between reduced lung compliance related to ARDS and ventilator dyssynchrony. 

Obtaining imaging was up to the discretion of the clinical team. Pneumothorax diagnosis may have been incidental, due to routine chest radiographs or POCUS, or may have been directed based on clinical suspicion, which was unable to be determined from this retrospective review. The clinical significance of an incidental pneumothorax is unclear. Moreover, a greater number of CT scans would have given a better insight into changes in lung architecture and any possible correlation with pneumothorax development. 

There are no established guidelines or criteria of when to pursue a conservative or an invasive approach to managing pneumothorax, both prior to and during the COVID-19 pandemic. Consequently, the choice of management was left solely up to the clinical team, and clinical reasoning was unable to be determined from this retrospective review. 

Our cases were also collected based on author recall at two hospitals, and as such, case-capture may not have been complete. We were also unable to access cases for the rest of the Northwell Health system, consisting of 21 other hospitals.

## Conclusions

We describe a case series of patients with COVID-19 associated ARDS who developed pneumothorax. Despite limitations, we adhered to best practice ventilator management in ARDS but still observed a large number of pneumothoraces during this pandemic compared to our pre-COVID-19 ARDS experience. Conservative management may be appropriate if there are no clinical signs or symptoms of tension physiology and pneumothorax size is small. 
